# Effect of multiple use and sterilization on sealing performance of bipolar vessel sealing devices

**DOI:** 10.1371/journal.pone.0221488

**Published:** 2019-08-20

**Authors:** Silvia Gardeweg, Barbara Bockstahler, Gilles Duprè

**Affiliations:** 1 Department for Small Animals and Horses, University of Veterinary Medicine, Vienna, Austria; 2 Clinic for Small Animal Surgery, Department for Small Animals and Horses, University of Veterinary Medicine, Vienna, Austria; Royal Infirmary of Edinburgh, UNITED KINGDOM

## Abstract

**Background:**

Advanced bipolar vessel sealing devices are widely used in human and veterinary medicine to reduce the operation time and intraoperative blood loss. Because most devices are made for a single use, their application is cost intensive. The aim of this study was to investigate the influence of multiple uses and sterilization on the performance of bipolar vessel sealing devices.

**Methods:**

The burst pressure of sealed porcine renal arteries was compared between the disposable devices „LigaSure”(Valleylab) and „Caiman“(Braun Vetcare) and the reuseable device „MarSeal”(KLS Martin). Additionally, the influence of the sterilization process was investigated, and the sealing time, number of cutting activations and tissue sticking were noted.

**Results:**

The disposable devices showed reliable performances over multiple activations and sterilization cycles, except for one Caiman device. Seals created with all devices achieved supra-physiologic burst pressures, with the highest pressure measured in a cycle using a MarSeal device.

**Discussion:**

During 25 activations with and without intermittent sterilization, no correlation was found between increasing seal numbers and decreasing burst pressure. However, the number of cycles in our study was limited to five.

**Conclusion:**

For limited numbers of cycles and sterilization procedures, seals created with the disposable vessel sealing devices LigaSure and Caiman achieved burst pressures comparaable to those produced with the reuseable MarSeal.

## Introduction

Bipolar electro-surgery relying on tissue response generators has experienced the most recent advancement in vessel sealing technology. These devices fuse vessel walls and create seals using a combination of electrical current and mechanical pressure. Collagen and elastin denature in the targeted tissue, and the mechanical pressure forms a coagulum. Depending on the instrument chosen, vessels up to 7 mm in diameter can be surgically sealed [1]. Shortly after their establishment in human medicine, bipolar vessel sealing devices gained increasing importance in veterinary surgery [2]. Suture ligation can be time-consuming and technically demanding, especially during laparoscopic procedures. Under these circumstances, bipolar vessel sealing devices are advantageous, because they help reduce intraoperative blood loss, the surgery duration and postoperative complications. Although small vessels with a diameter of 1–2 mm can be reliably sealed by monopolar coagulation, bipolar vessel sealing devices are able to seal vessels with diameters of 5 mm or greater. These devices create strong seals by targeted, feedback-controlled delivery of compression and heat. Thermal damage of adjacent tissue is reported to be limited to a 1–2 mm section [3]. The vessel sealing strength is defined via the burst pressure [4]. This value describes the maximum intraluminal pressure achieved before leakage occurs at the sealing site. For burst pressure testing, typically physiologic saline is infused ex vivo into sealed vessel sections using a computer-controlled syringe-pump with an integrated pressure transducer [5]. Although reduction of the surgical duration and fewer postoperative complications are economically advantageous factors, the use of bipolar vessel sealing devices is rather cost-intensive. The most commonly used vessel-sealing instruments, such as LigaSure® and Caiman®, are designed for a single use. MarSeal® is an exception, because it is intended for use in more than one surgery. Although many veterinary surgeons do “reuse” those devices, the effect of multiple uses and sterilization on the sealing performance of these different bipolar vessel sealing devices is unknown. Therefore, the aim of this study was to compare the seal failures and maximum bust pressures of 2 disposable devices (Caiman and LigaSure) with those of the reuseable device MarSeal over multiple seals with and without subsequent sterilization. Our hypothesis was that the LigaSure and Caiman devices, which are intended for single use only, would show a decreasing ability to perform safe seals over multiple operations and sterilizations compared to that of the reuseable MarSeal.

## Materials

### Vessel sealing devices

**Fig 1:**
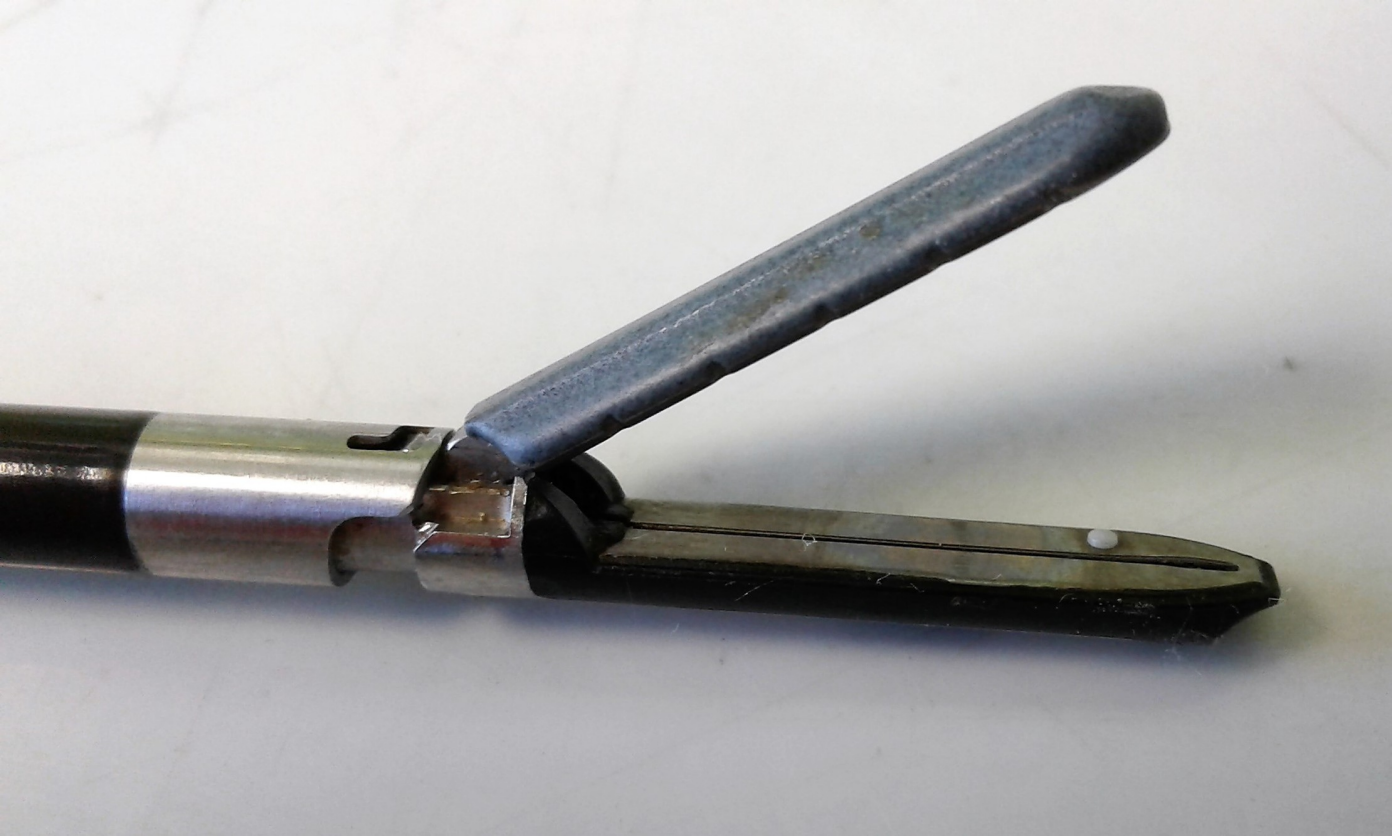
MarSeal jaw configuration.

**Fig 2:**
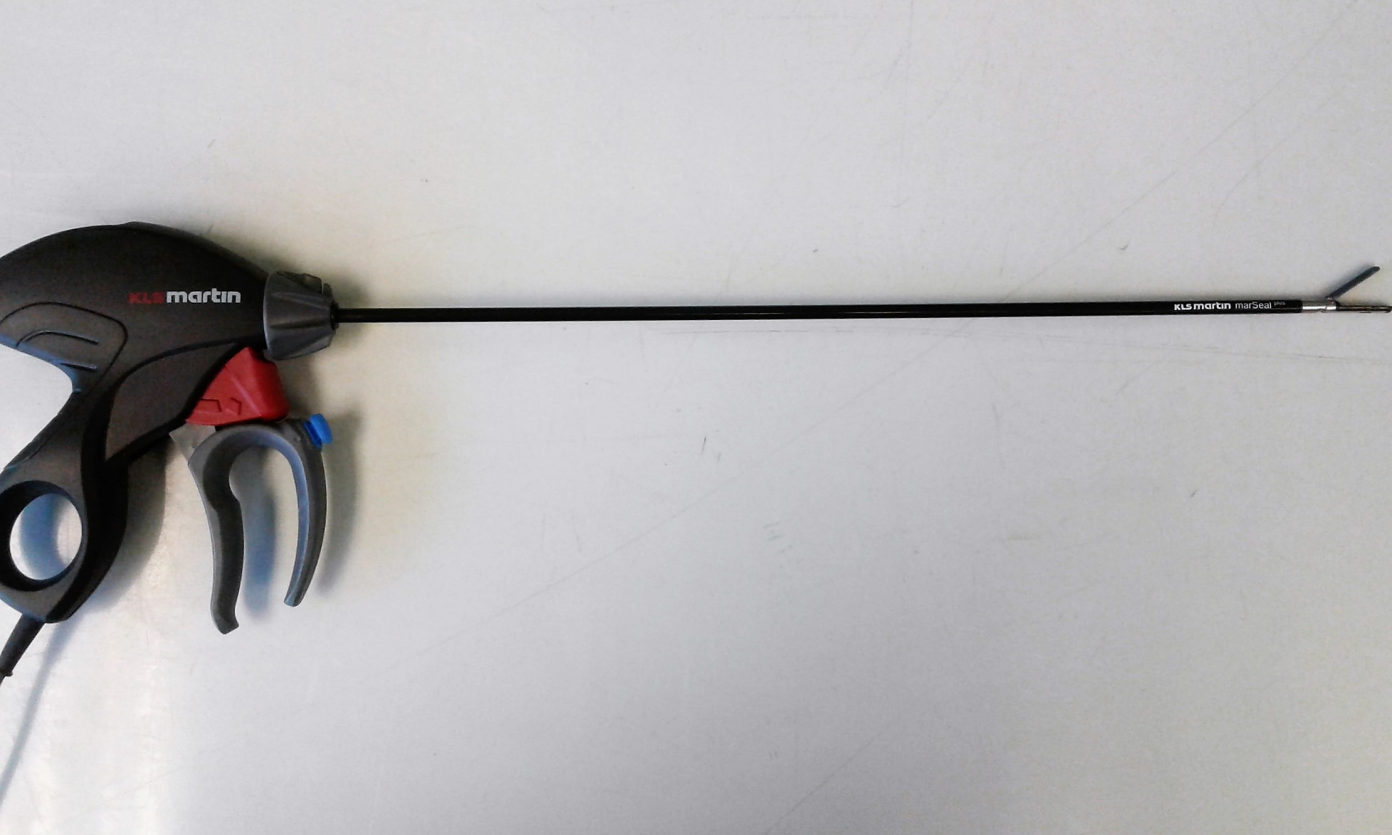
MarSeal device.

The MarSeal 5 plus (KLS Martin, 78532 Tuttlingen, Germany) is a reuseable vessel sealing device, with a 37-cm shaft length that is, intended for laparoscopic surgery. The manufacturer warrants a reliable sealing performance for a minimum of 50 routine operations. Veins, arteries and tissue bundles up to 7 mm in diameter can be occluded and cut after seal completion. All parts of the instrument can be taken apart and autoclaved at 134°C except for the blade [6]. The bipolar vessel sealing system consists of the bipolar MarSeal instrument, which is shown in Figs 1 and 2, and the high-frequency generator, which offers a high power-to-voltage-ratio (with a maximum permissible HF voltage of 3000 Vp). The generator continuously measures the impedance of the target tissue during the sealing process. The impedance changes due to contribution of the high-frequency current. The G-level represents the impedance threshold at which the seal is considered complete. At G1, the impedance level is low and the sealing process short. G3 is the default setting and, provides a medium impedance threshold. At G5, the impedance threshold is high, resulting in a longer sealing process.

**Fig 3:**
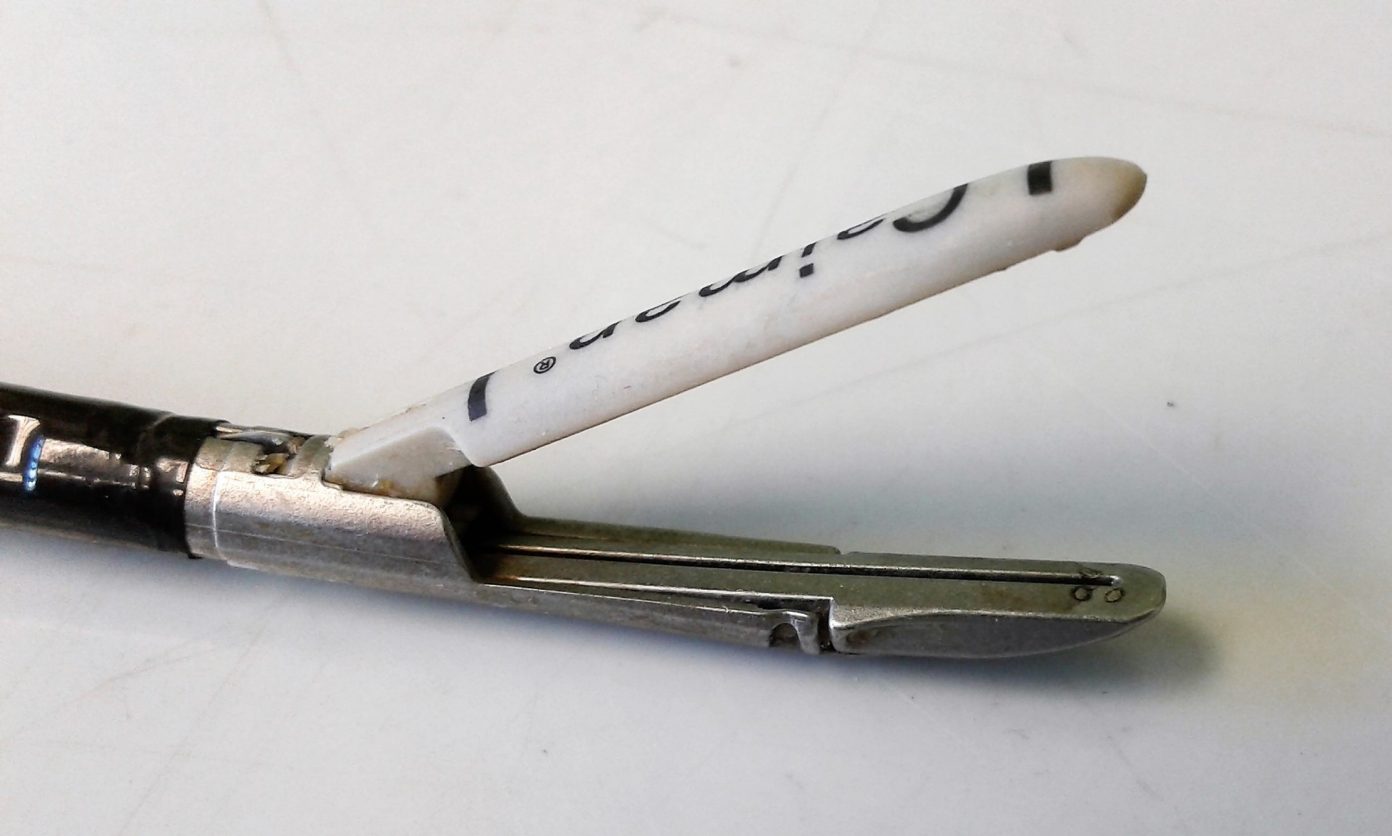
Caiman jaw configuration.

**Fig 4:**
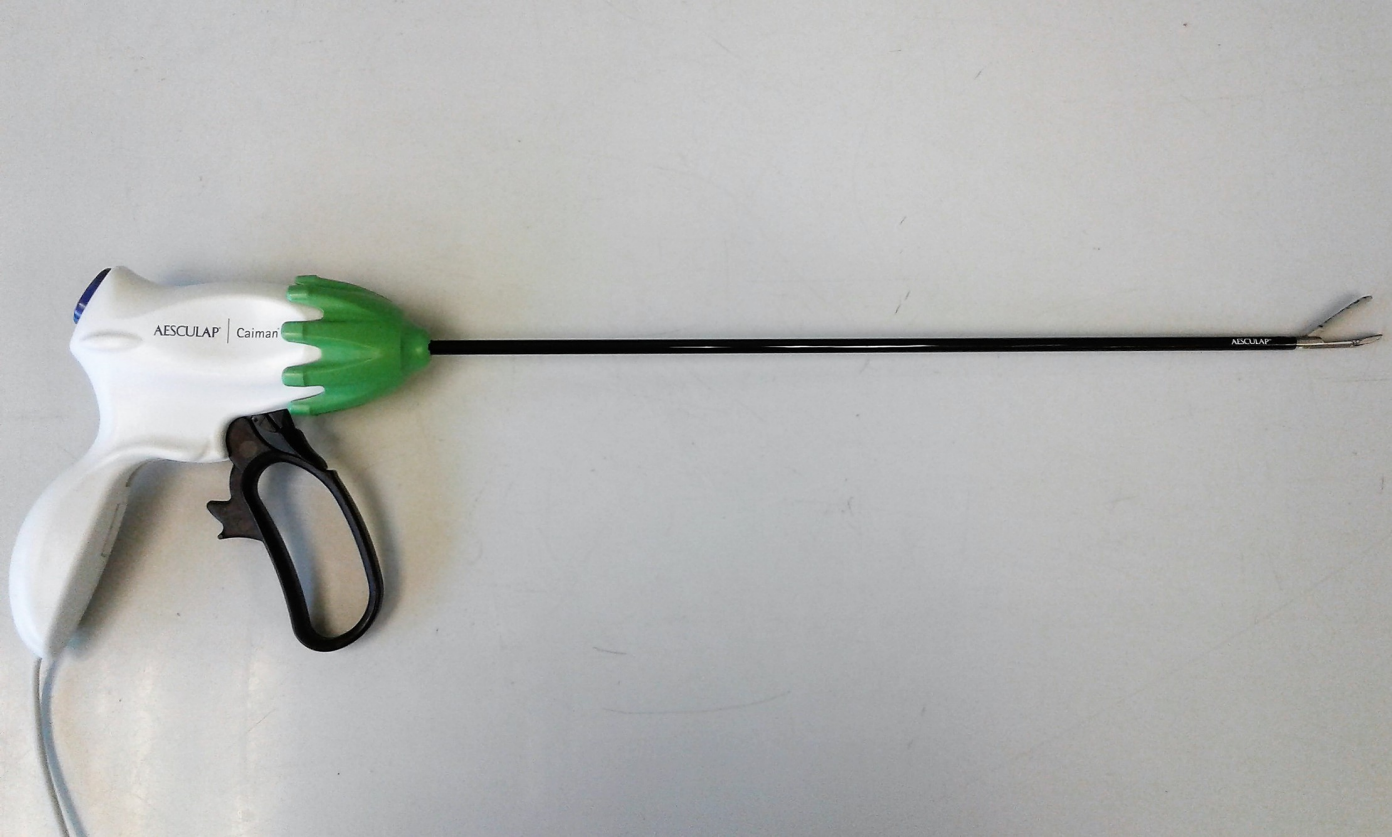
Caiman device.

The Caiman 5 (Braun Vetcare, 78532 Tuttlingen, Germany) instrument is a single-use component of the Aesculap Seal and Cut Technology and is only compatible with the Lektrafuse RF Generator, which supplies a maximum voltage of 240 Vp [7]. The device creates a seal by applying bipolar electrosurgical energy to a vessel interposed between the jaws, as shown in Fig 3. Included in the instrument shown in Fig 4 is a cutting blade for tissue division. Caiman offers two different settings: standard mode and power level. The impedance, voltage and current are monitored by the Lektrafuse software. According to the manufacturer’s advice, the sealing duration and power setting do not require any user adjustment.

**Fig 5:**
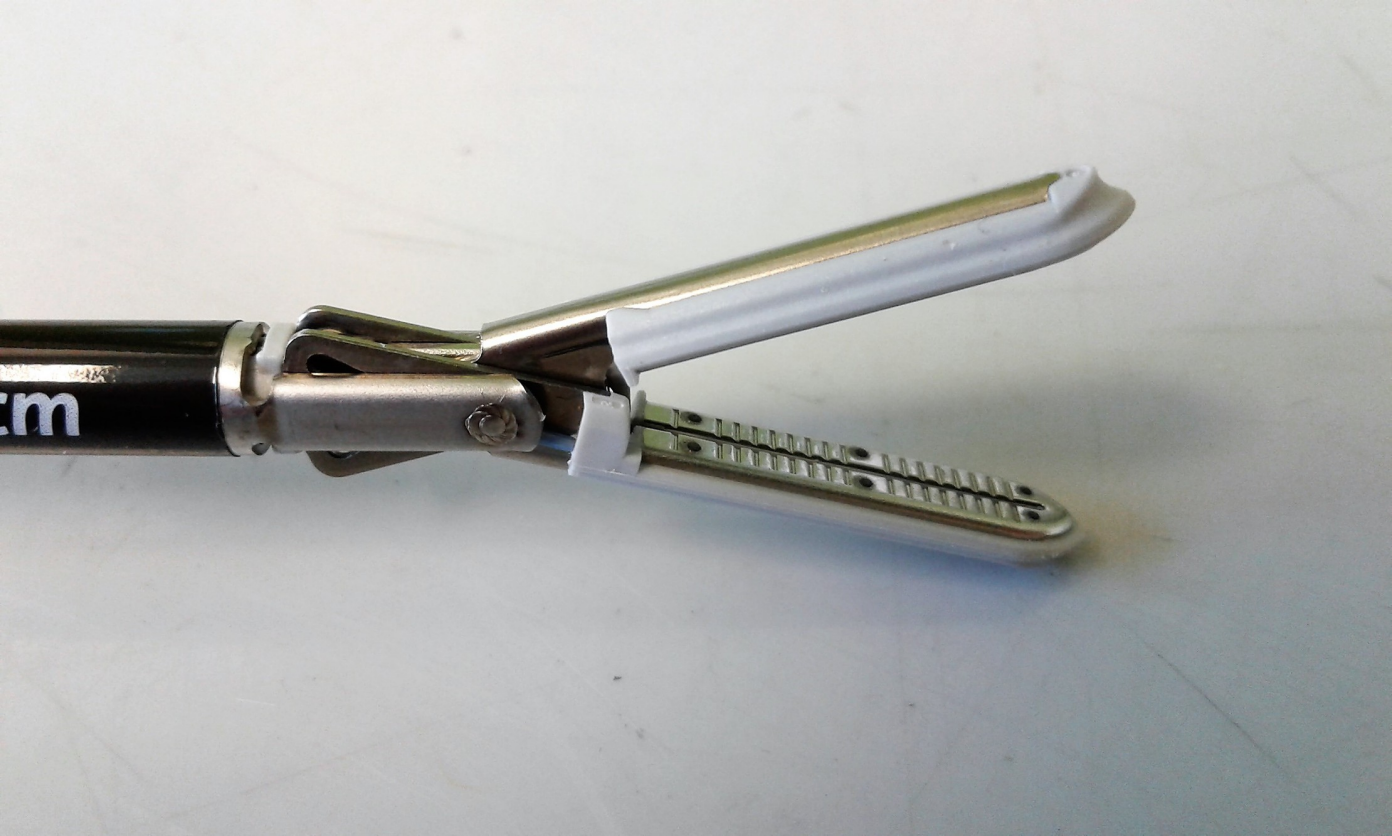
LigaSure jaw configuration.

**Fig 6:**
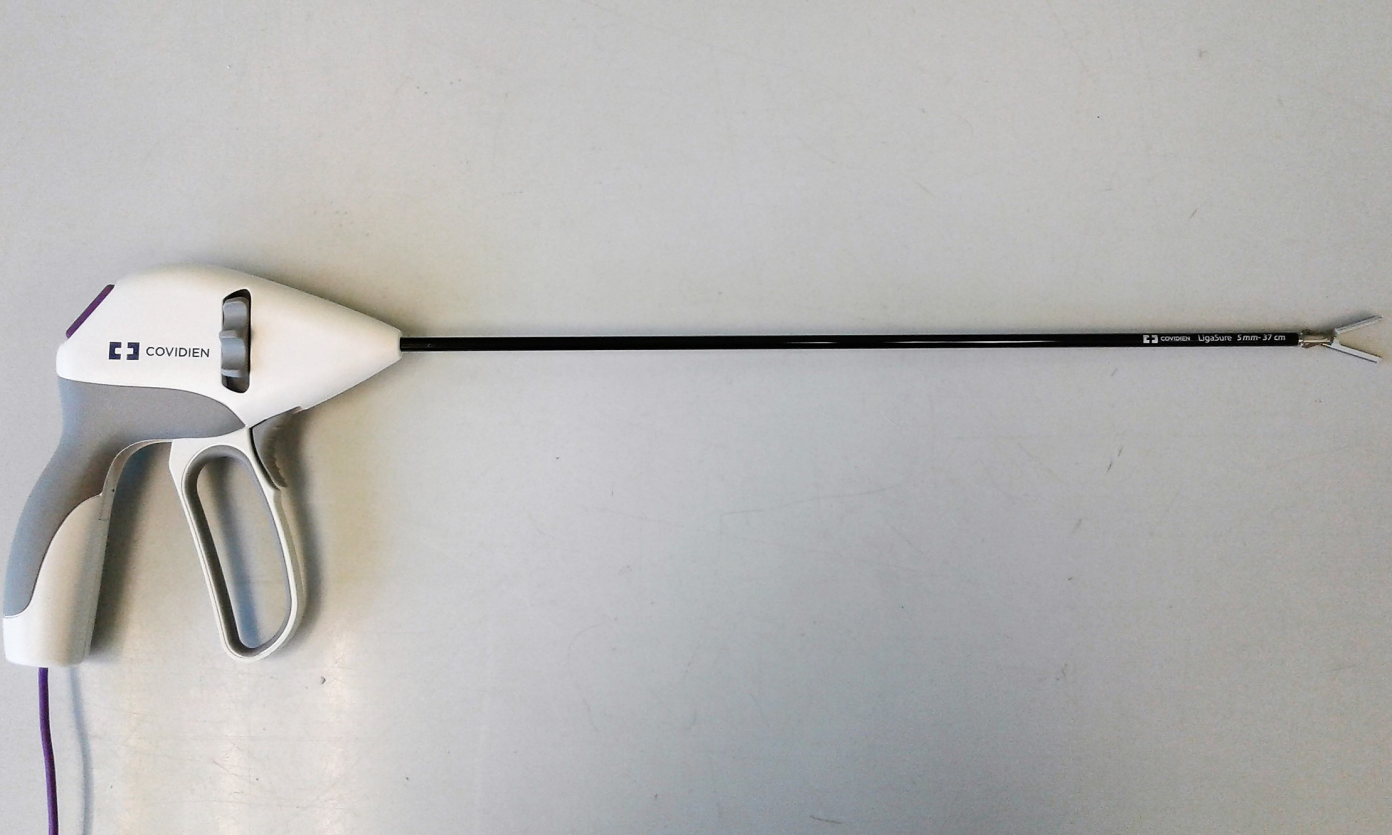
LigaSure device.

The LigaSure 5-mm blunt tip (Medtronic, Minneapolis, MN 55432–5604, USA) is a bipolar vessel sealing device with sealing and cutting capabilities that is designed for a single use. The LigaSure jaw configuration is shown in Fig 5 and the device in Fig 6. The Force triad energy platform provides energy for monopolar and bipolar surgical applications featuring three touchscreens. Three energy levels can be chosen: one bar, two and three bars. These bars represent different desiccation levels and allow a precise effect on the target tissue. The standard setting is two bars. By selecting one bar, the application of heat and pressure is reduced compared with that of the two and three bar levels, but the sealing process may be extended. Three bar fusion cycles result in a higher output voltage, which is more effective in thicker tissues [8].

### Experimental protocol

#### Devices

**Fig 7:**
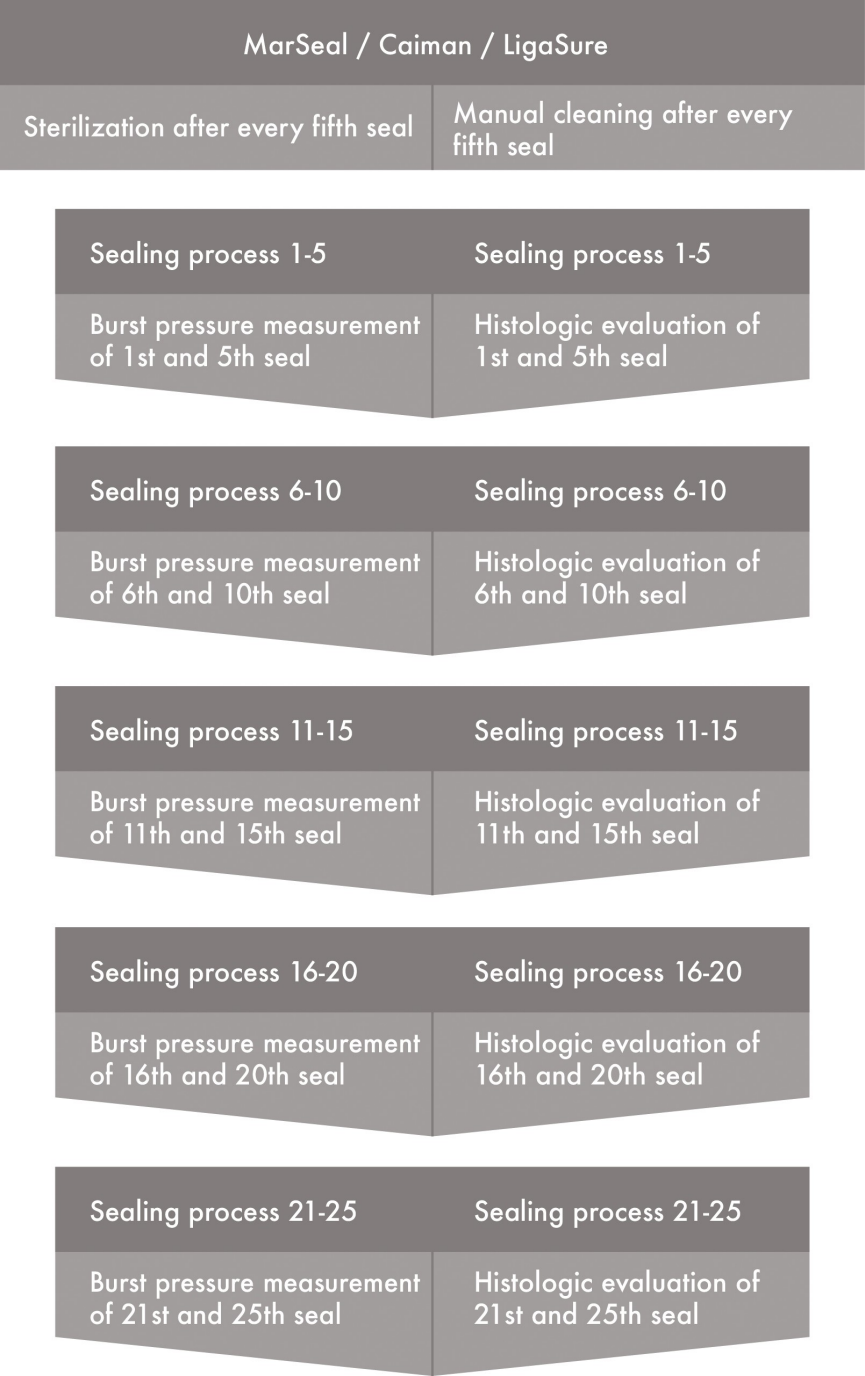
Flow chart of the experimental procedure.

For this experimental study, each device performed 25 seals in total. If the process included sterilization, then the device was sterilized after every fifth seal. This procedure is summarized in a flow chart in Fig 7.

**Fig 8:**
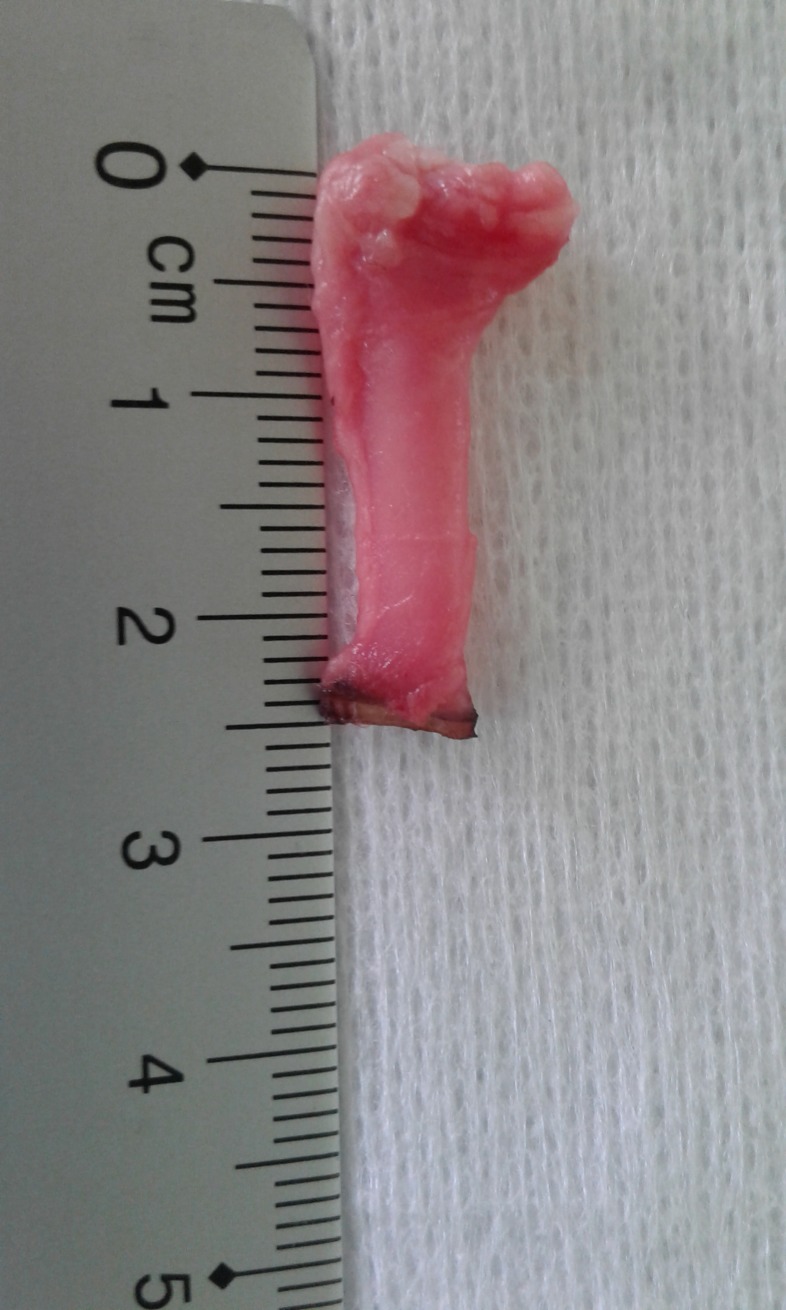
Sealed renal artery.

The “hand pieces” were cleaned manually when sterilization was not included. Only seals with numbers 1, 5, 6, 10, 11, 15, 16, 20, 21 and 25 were evaluated for burst pressure. The numbers and types of instruments are presented in Fig 8. the Caiman and LigaSure were tested using two hand pieces for the cycles with and without sterilization. For the Caiman, all seals were performed in standard mode. For the LigaSure instrument type, the setting was three bars. However, each cycle was repeated at the two-bar level using one device each. For the MarSeal, all cycles were performed using the same hand piece, in G3 and G5 modes. After every fifth seal, the devices were ether cleaned manually and subsequently sterilized (“steril” in Table 1) or only cleaned (“nonsteril” in Table 1).

**Table 1:** The selected test and energy modes of the instruments.

	Caiman		LigaSure				MarSeal			
	Steril	Non-steril	Steril	Steril	Non-steril	Non-steril	Steril	Steril	Non-steril	Non-steril
e	Standard	Standard	3 bar	2 bar	3 bar	2 bar	G3	G5	G3	G5
n	2	2	2	1	2	1	same unit for all procedures			

e = energy mode chosen, n = number of devices

#### Renal arteries

**Fig 9:**
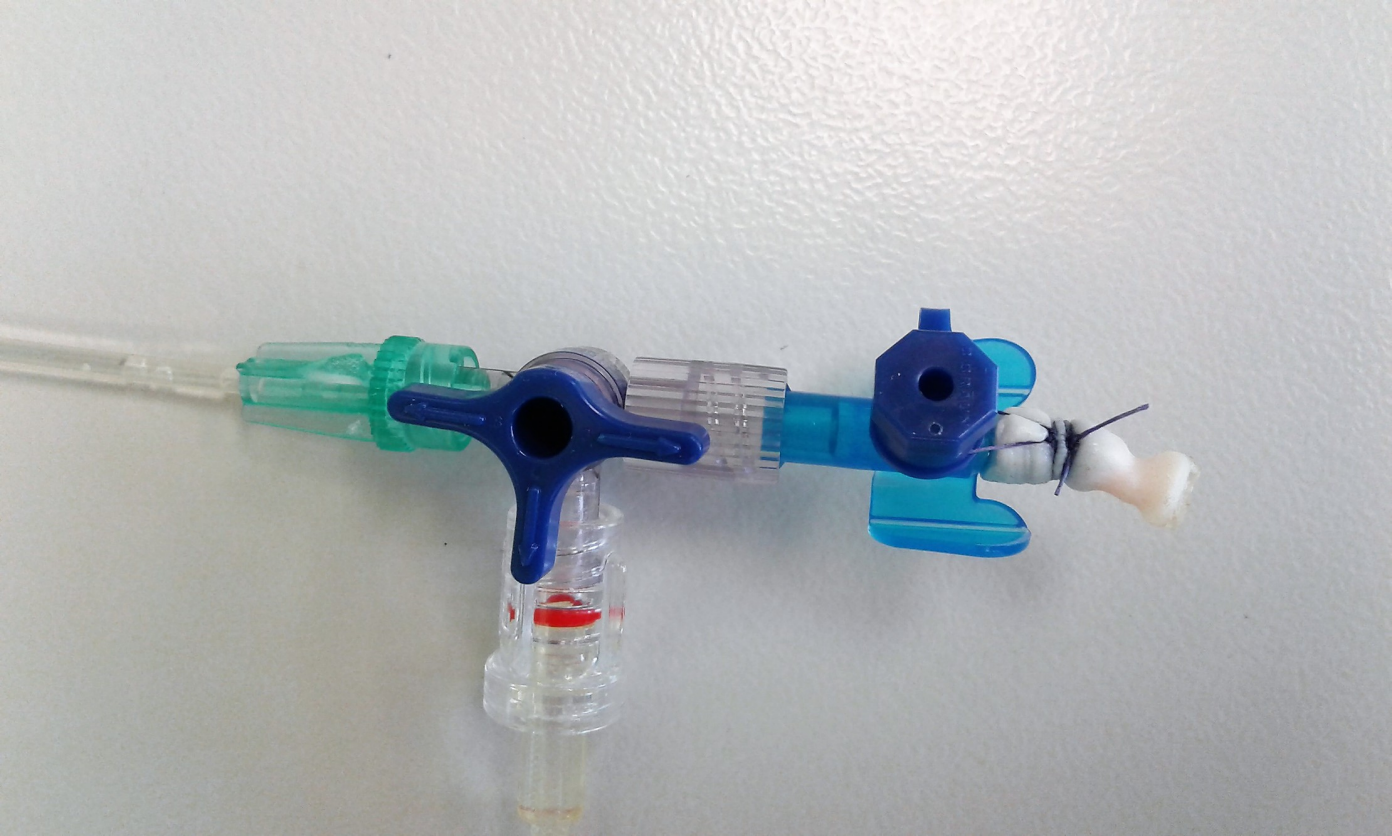
Sealed arterial segment attached to a venous catheter.

All seals were performed using porcine renal arteries with an outer diameter ranging from 4.1 mm to 7.32 mm for LigaSure, 4.05 mm to 7.61 mm for Caiman and 3.42 mm to 7.71 mm for MarSeal. An example of a sealed renal artery is shown in Fig 8 and a sealed arterial segment attached to a venous catheter in Fig 9. To preserve the full length of the vessels and preserve injury, the kidneys were eviscerated from the carcasses with the surrounding fat and soft tissue, and the arteries were cut at their origin. After careful preparation of the arteries and removal of adjacent tissue, the arteries were transferred inside a portable cooling box and kept moistened with fresh water.

## Evaluated parameters/ Methods

**Fig 10:**
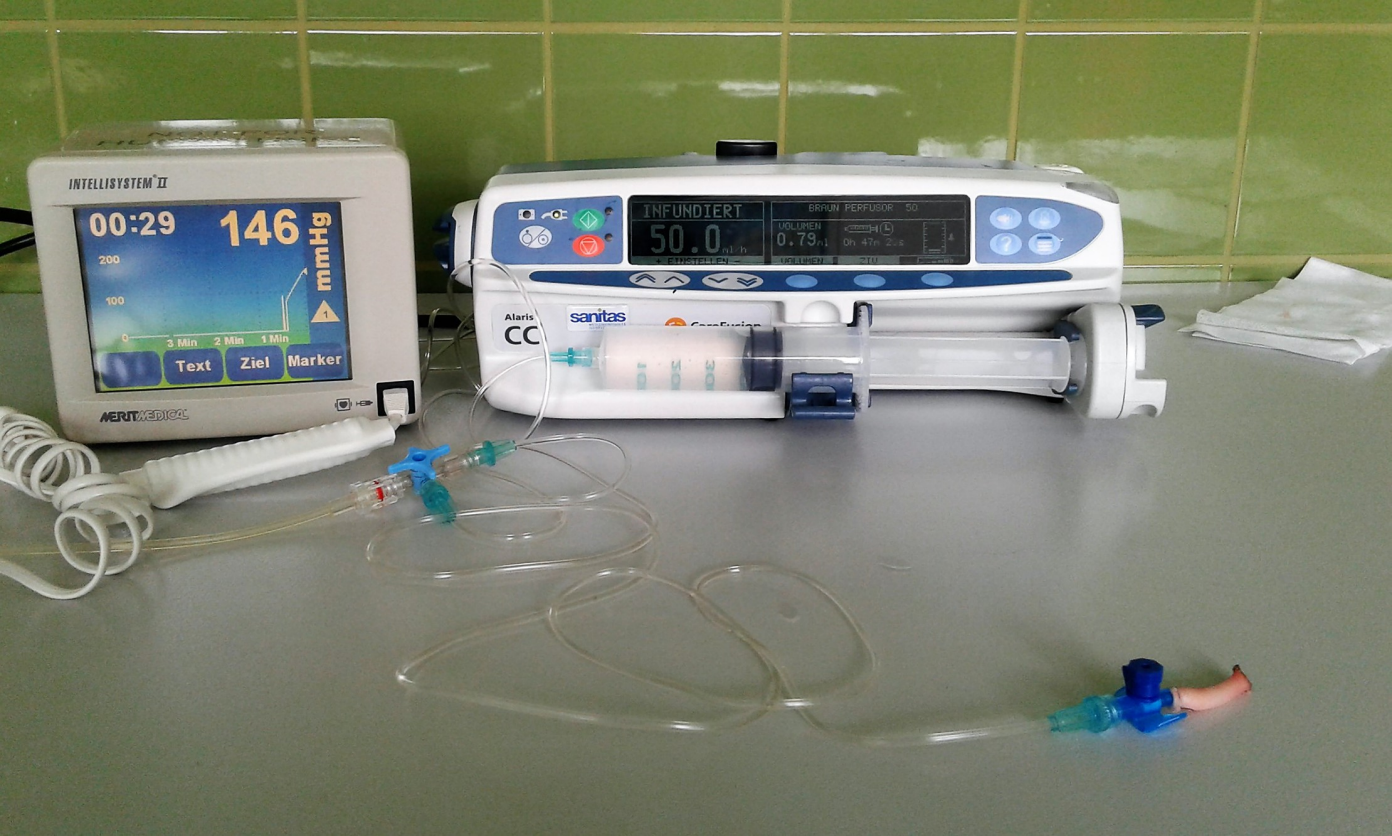
Experimental setup. The pressure transducer (Intellisystem II, Merit Medical) is on the left, the infusion pump (Alaris Care Fusion) is on the right, and the attached arterial segment is in the front.

Following measurement of the diameter and length with a digital caliper, the arteries were sealed using the tested instruments. The mean length of a sealed segment was 24.21 mm. For burst pressure testing, a 22-gauge venous catheter was introduced into the open end of the vessel and secured using Vicryl 3/0 suture material (Coated Vicryl, Polyglactin 910 Suture, Ethicon USA, LLC. 2017. 085133–171129). The experimental setup is pictured in Fig 10. After placing the arterial segment between the instrument jaws, sealing was performed until an audio signal produced by the generator confirmed seal completion. Subsequently, the cutting mechanism was activated. The number of cutting activations necessary to transect each artery was counted, and the severity of tissue sticking was evaluated subjectively on a scale from 0 for no sticking to 3 for severe sticking. All seals were performed by the same person. The sealing time was recorded using a digital stopwatch. Physiologic saline was infused at a fixed rate of 50 ml/h using a syringe pump (Alaris Care Fusion), and the intraluminal pressure was recorded with a pressure transducer (Intellisystem II, Merit Medical). The integral guard prevented infusion by the syringe pump at an intraluminal pressure higher than 900 mmHg (+ / - 5%). When an alarm sound was produced by the syringe pump, indicating high intraluminal pressure, the syringe was removed from the pump and the infusion was continued manually. A burst was visible as leakage at the sealing site and a massive drop of the intraluminal pressure within a fraction of a second. In cases with leakage from a different site, the vessel was excluded from the subsequent analysis. The tested bipolar devices were sterilized using a low temperature sterilizer (FA 95, Webeco Matachana Group, Barcelona, Spain). The Instruments passed a low temperature steaming process with formaldehyde as an active agent. The formaldehyde content of the steam gas mixture is only 2%, because the OH-group product of formaldehyde and water has higher reactivity towards organic material in the gaseous state. Penetration and inactivation are improved, and microbial cells are inactivated by nucleic acid methylation and protein denaturation. The modified steam sterilization cycle of approximately four hours consists of three parts. The process starts with a pulsed injection of formaldehyde steam, alternating with the vacuum stages. The pressure increases during this procedure to ensure that the sterilizing agent reaches all surface areas of the treated devices. During the second phase, the pressure is kept constant in the sterilization chamber via a barometric adjusted pressure control. Formaldehyde must be available at a defined concentration for effective microbicidal interactions. In the last phase, air is evacuated from the sterilization chamber. Pulses with pure water steam remove the sterilizing agent and the components of the chemical reaction from the devices. After vacuum drying and aeration phases, the program is completed.

For the Caiman device, all seals were performed in standard mode according to manufacturer’s advice. The burst pressure was determined for 20 arteries each sealed during cycles including and not including sterilization of the device. For the LigaSure, the burst pressure was measured for 20 seals from cycles including and not including sterilization with the performing device working in the three- bar mode. Subsequently, the burst pressures of 10 seals from the sterilization and nonsterilization cycles produced using the two- bar mode were also measured. For the MarSeal, the burst pressures were evaluated for 20 seals each created in G3 mode with and without hand piece sterilization. Additionally, the burst pressures were evaluated for 10 seals each created in G5 mode with and without hand piece sterilization.

### Burst pressure

Burst pressure is defined as the highest intraluminal pressure before a burst and is an essential criterion when defining whether a seal is considered successful or not. In accordance with recent studies [2, 9], the minimum burst pressure for successful seal of an artery was determined to be 300 mmHg. This value is more than twice as high as the blood pressure of healthy animals. In their 2008 study, Wallwiener et al. defined 250 mmHg as the minimum intraluminal pressure that must be withstood by a successful seal [9]. The influence of the sterilization on the sealing performance of the disposable Caiman and LigaSure devices was tested compared to that of the reuseable MarSeal device. The possible influence of the energy mode was also investigated.

### Statistical analysis

Recorded data were entered into an Excel spreadsheet (Microsoft Office 2010). The impacts of the factors “device tested” and “sterilization” on the measured burst pressure value were analyzed using a general linear model. The comparison between devices was performed using Bonferroni’s alpha correction procedure. Furthermore, we investigated the development of burst pressure as a function of increasing sealing numbers by linear regression analysis. For all analyses, a p-value < 0,05 was considered significant.

## Results

### Burst pressure

**Fig 11:**
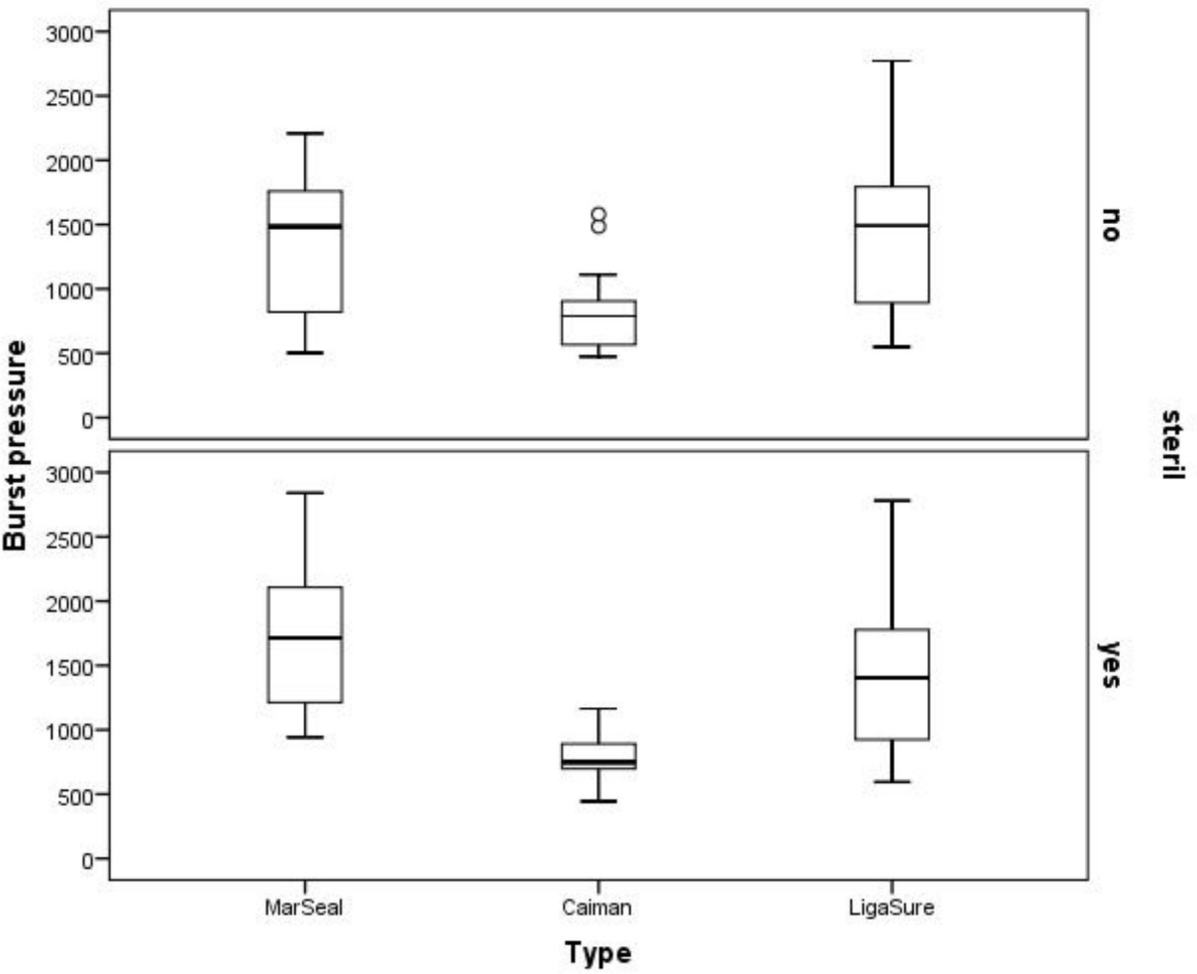
Box plot diagram of all measured burst pressures. MarSeal: grey box plot, Caiman: red box plot, and LigaSure: green box plot. The burst pressure is plotted on the y-axis, and the instrument type is plotted on the x-axis.

Supra physiologic burst pressures were achieved for all completed seals. One Caiman instrument was unable to complete the last sealing session during one cycle with sterilization. The failure was detected by the generator itself, which gave the information that user intervention was needed. Altogether, the measured burst pressures were higher than the defined safety index of 300 mmHg. All measured burst pressure values are represented in Fig 11 in a box plot diagram.

The maximum burst pressure for an artery sealed with the MarSeal device was 2841 mmHg. This device underwent a cycle without sterilization and was at the G3 level. The diameter of the sealed vessel was 4.28 mm, and the sealing time was 7.71 seconds. This burst pressure was achieved during the 21^st^ seal of the series and was the highest burst pressure measured during the whole experiment. The minimum burst pressure for an artery sealed with MarSeal was 649 mmHg. This pressure occurred during the 10^th^ seal of the device, which was working at the G3 level in a series without sterilization. The sealing time was 5.94 seconds, and extended smoke production was noticed. The median burst pressure for arteries sealed with MarSeal was 1545 mmHg. The maximum burst pressure for an artery sealed with Caiman was 1580 mmHg. This value was obtained during the 10^th^ seal of the series, and it took 6.92 seconds to seal an artery 6.31 mm in diameter. The minimum burst pressure for an artery sealed with Caiman was 445 mmHg. This was the lowest burst pressure measured during the experiment. This value was obtained during the 10^th^ seal of a cycle including sterilization. The device used showed irreversible damage to the clamp after the fourth sterilization and was not able to perform the 20^th^ seal. Arteries sealed with Caiman withstood a median burst pressure of 764 mmHg. The maximum burst pressure for an artery sealed with LigaSure was 2781 mmHg. The device worked at the “three bars” level during a cycle that included sterilization. The diameter of the sealed artery was 6.00 mm and had a sealing time of 5.49 seconds. The minimum burst pressure for an artery sealed with LigaSure was 550 mmHg. The device worked at the “two bars” level, and it was the first seal of a cycle that included sterilization. It took 4.32 seconds to seal an artery 4.63 mm in diameter. For arteries sealed with LigaSure, the median burst pressure was 1446 mmHg.

#### Performance over multiple operations

**Fig 12:**
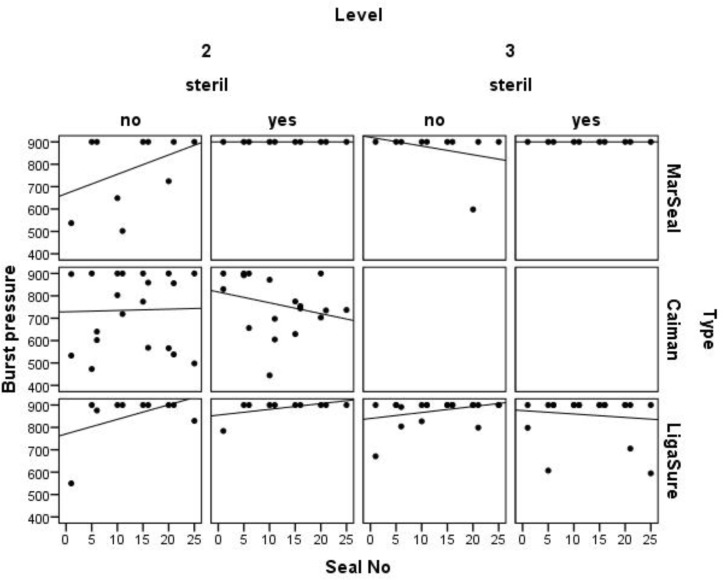
Scatter diagram of all measured burst pressures as a function of the seal number. The burst pressure is plotted on the burst pressure is plotted on the y-axis and the seal number is plotted on the x-axis. Curve: the regression grade indicates a tendency for increasing or decreasing burst pressure.

Burst pressure values, produced with the MarSeal in G3 mode, the LigaSure in the two- bar mode and the Caiman in standard mode, were measured during cycles without sterilization and showed non-significant changes during the experiment. Conversely, the detected regression grade for the burst pressures of seals produced with Caiman during cycles including sterilization, decreased. The burst pressures of seals produced with the MarSeal in G3 and G5 modes during cycles both including and not including sterilization appeared constant in this presentation and were all greater than 900 mmHg. The burst pressures of seals created with the LigaSure in the highest energy mode during cycles not including sterilization showed a rising tendency. Considering the burst pressure values measured after 25 sealings, we found no significant decrease in the burst pressure with an increased number of sealings regardless of the instrument type tested (MarSeal vs. Caiman–p = 0.4, Caiman vs. LigaSure–p = 0.78, LigaSure vs. MarSeal–p = 1.0). Fig 12 shows the measured burst pressure values as a function of the seal number.

**Fig 13:**
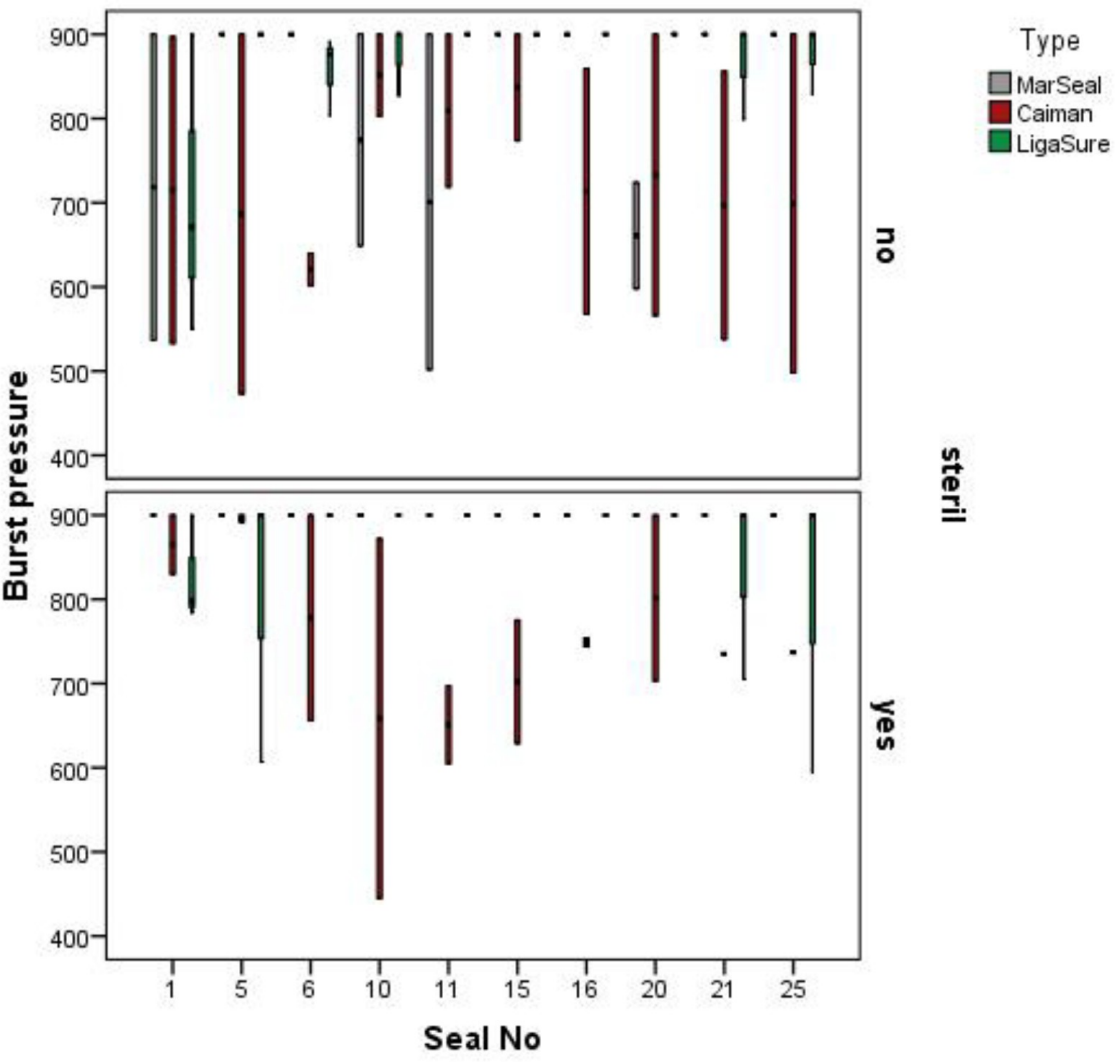
Measured burst pressures of seals created with the MarSeal in G3 mode, Caiman in standard mode and LigaSure at the two- bar level. The box plot is colored gray for MarSeal, red for Caiman and green for LigaSure. The burst pressure is plotted on the y-axis, and the instrument type is plotted on the x-axis.

To identify potential differences that could be traced back to the energy levels, all measured values were separated based on the energy mode. The burst pressure produced using the G3 mode for the MarSeal, two bars for the LigaSure and the standard mode for the Caiman are pictured in the upper section of Fig 13. The lower section represents all values measured for seals produced with the LigaSure using the three- bar mode and the MarSeal using the G5 mode. When the measured burst pressure values were compared, no significant difference was detected between the two energy modes for the LigaSure and MarSeal.

#### Influence of sterilization

**Fig 14:**
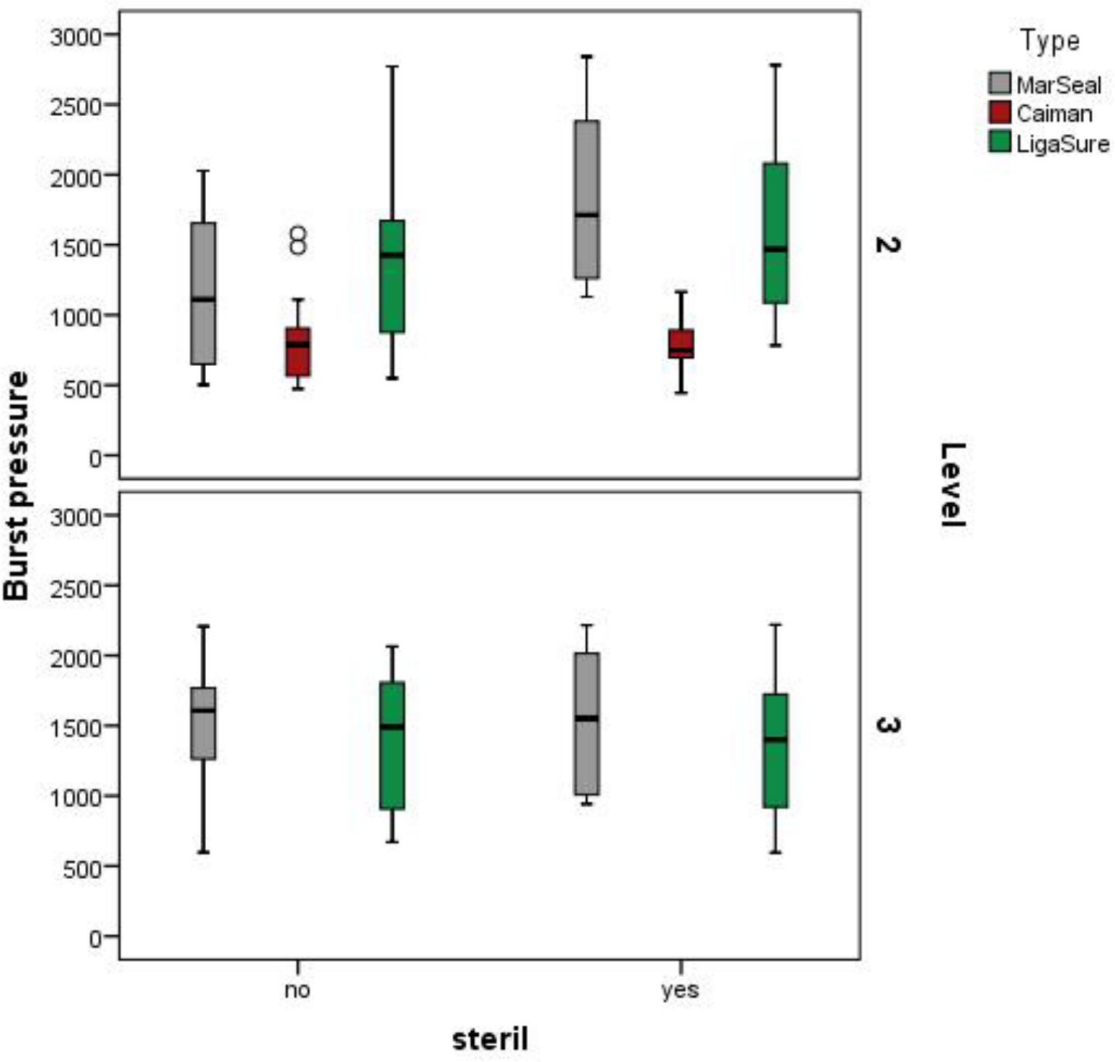
Range of burst pressures during cycles with and without sterilization. The box plot of the results is shown in gray for MarSeal, red for Caiman and green for LigaSure.

The influence of sterilization was investigated by comparing each instrument with itself. The range of burst pressures during cycles with and without sterilization are shown in Fig 14.

During cycles not including sterilization, the maximum burst pressure achieved for a seal produced using the MarSeal device was 2841 mmHg, and the minimum burst pressure was 649 mmHg. The median burst pressure for arteries sealed with MarSeal during cycles without sterilization was 1492 mmHg. The maximum burst pressure for a seal produced with the MarSeal device during cycles including sterilization was 2384 mmHg; the minimum burst pressure was 941 mmHg, with a median of 1713.5 mmHg. The maximum burst pressure measured for a seal produced with the Caiman device during a cycle not including sterilization was 1191.50 mmHg; the minimum value was 621 mmHg. 1028 mmHg was the maximum burst pressure measured for a vessel sealed using a Caiman device during a cycle including sterilization; the minimum value detected was 651 mmHg. During cycles not including sterilization, the median burst pressure was 788.5 mmHg for arteries sealed with Caiman; for cycles including sterilization it was 749 mmHg. The maximum burst pressure achieved for an artery sealed using the LigaSure device during a cycle not including sterilization was 2771 mmHg; the minimum burst pressure measured amounted 550 mmHg. During cycles including sterilization, the maximum burst pressure achieved using the LigaSure device was 2781 mmHg; the minimum burst pressure detected was 784 mmHg. During cycles not including sterilization, the median burst pressure was 1492.5 mmHg, and for cycles which included sterilization it was 1405 mmHg.

## Discussion

The primary aim of this study was to test the vessel sealing quality of one reuseable and two disposable vessel sealing devices measured by the burst pressure, which defined the efficacy of vessel sealing. Specifically, we aimed to compare the performance of these devices over multiple operations and with or without re-sterilization. We found that the LigaSure devices were able to perform five subsequent cycles of five activations each without seal failure. Sterilization proved to have no hazardous impact on sealing performance, as the lowest burst pressure detected during a cycle including sterilization was 784 mmHg. Decreasing burst pressure values were not noted over five cycles, and blade function showed no signs of wear. Sticking and charring occurred, but they did not change considerably throughout the experimental procedure. Thus, the performance of the LigaSure was comparable to the objective standard, MarSeal. The lowest burst pressure was measured for a seal performed on an artery measuring 5.80 mm in diameter as the 10^th^ seal of a Caiman device that had been sterilized once. This lowest burst pressure still reached 445 mmHg, which was more than three times the physiologic blood pressure. The same instrument showed irreversible clamp damage after the fourth sterilization and was not able to perform the 20^th^ seal. It is important to note that the instrumental failure was identified by the generator, which produced an alarm signal, that required manual intervention. This alarm would have prevented unnoticed hemorrhage. As the incident was accompanied by extensive smoke production, visibility might have been altered in real laparoscopic surgery. Sticking and charring were not disruptive or increased while using Caiman devices over multiple operations. Burst pressure values measured during cycles including and not including sterilization showed non-significant tendencies throughout the repetition cycles. Regarding the results for the seal reliability of the LigaSure devices, our findings reflect the results of Kuvalinda et al. (2019) [10], who tested potential reuse of LigaSure Small Jaw and LigaSure Impact. They performed simulated canine splenectomy and subsequent burst pressure testing on porcine carotid arteries and arrived at the conclusion that reuse and re-sterilization resulted in seal failure after ten or more cycles. In the study of Valenzano et al. (2019) [11] 5-mm LigaSure Maryland jaw devices were tested on canine cadaver ovariectomy models. They revealed seal failure after a minimum of 4 and a maximum of 12 cycles. In contrast to our findings and those of Kuvalinda et al., failure of the activation button appeared to be the main reason for seal failure. This observation was not noted in the present or other concurrent studies [10, 12]. The Caiman was the device that performed the seal withstanding the lowest intraluminal pressure. This result reflects the observation of Kuvalinda et al., which showed that devices creating seals withstanding comparatively low intraluminal pressure might provideworse performance, thus leading to seal failure or breakage of the device.

In our study, the clamps of each device were cleaned manually after every fifth seal. The absence of increasing charring was possibly prevented by the cleaning method, as the clamps were only touched using moist cotton swabs to not damage their surface. These finding correlate with those of Blake et al. 2017 [12], in which the influence of reuse and resterilization on the performance of the LigaSure Atlas was evaluated.

Although the burst pressure is directly correlated to the performance of a bipolar vessel sealing device, it is also influenced by several factors other than the instrument itself. Currently, the diameter of vessels that can be safely sealed using bipolar vessel sealing devices is limited to 7 mm. Bibi et al. tested the ability of the EnSeal® device (Ethicon), which is a compareable bipolar vessel sealing device, to seal vessels up to 14 mm in diameter. The authors observed a significant decrease in burst pressure, which was negatively correlated with the vessel diameter. In that study, the median burst pressure for arteries with a median diameter of 14 mm was 85 mmHg with a 31% failure rate [13]. Although the EnSeal was not tested in our study, these results suggest that the vessel diameter plays a major role and that manufacturer restrictions should be applied cautiously. In our study, the mean outer vessel diameter was 5.36 mm, and all tested devices were approved for sealing vessels of this size. We do not know, how the vessel sealing devices tested in our study, would have performed on vessels of greater diameters than the current manufacters recommendations. In clinical setting, sealing is not always performed in a perpendicular manner, which increases the distance to be sealed. Moreover, vessels sealed perpendicularly have been shown to reach higher burst pressures than those sealed at an angle [14]. In our study, the ex vivo approach allowed all arteries to be sealed perpendicularly, which removed the impact of this artifact. The maximum burst pressure measured during our experiment of 2841 mmHg was produced with a MarSeal device as the 21st seal of a series of 25 seals with sterilization of the instrument after every 5th seal. Use of the same vessel type with similar sizes allowed direct comparison between the 3 devices. Klar et al. compared the LigaSure to the MarSeal using a sheep model. The femoral and carotid arteries were sealed in vivo, and the sealing time and failure rate were noted. Burst pressure was measured subsequently. In this study, the MarSeal failure rate was 7%, and the LigaSure failed in 9% of seals. The mean burst pressures were 429 mmHg and 484 mmHg, respectively [15]. The differences with our findings might be explained by the in vivo approach and the different artery type. Fusing collagen-containing vessel walls is the key mechanism in bipolar vessel sealing. D. Sindram et al. investigated the influence of collagen content on the seal quality using the LigaSure Atlas by sealing different types of porcine arteries in vivo [3]. The authors revealed significant differences in the burst pressures of the seals based on the collagen-elastin ratio and vessel strength, which correlated with the collagen-elastin ratio. Because we sealed only porcine renal arteries, a consistent collagen-elastin ratio was provided throughout our entire ex vivo study.

The effect of contamination on the sealing performance of a bipolar vessel sealing device was investigated by Wallwiener et al., who coated the jaws of another vessel sealing device, BiClamp® (Erbe), with blood, fat and collagen, before sealing porcine renal arteries. In that study, the sealing performance was not influenced by residual tissue sticking to the instrument jaws [9]. These results correlated with our observation, that vessels sealed with freshly cleaned jaws did not reach higher burst pressures than seals that were produced with jaws that had not been cleaned after performing multiple seals.

In our study, reuse of the LigaSure device up to five times did not result in an increased risk of seal failure nor in signs of wear. It must be noted that the breakage of one Caiman device during the experiment was preceded by smoke production and a detectable drop in performance. Clinically, this suggests that excessive smoke production could be a sign for a defective device. In the current study, sterilization using the formaldehyde gas sterilization process had no impact on the sealing performance of five instruments performing 125 seals. Out of six instruments undergoing cycles including sterilization, one Caiman device showed irreversible damage of the clamp after sterilization for the fourth time. During the sterilization process, instruments are exposed to a 2% formaldehyde steam-gas mixture. The OH-group of formaldehyde has a higher reactivity towards organic material in the gaseous state, and thus inactivation of pathogenic material is improved. Penetration of molecules may damage devices, that are only distributed for a single use. Similar to the results of these studies, no correlation was found between an increasing sealing number and decreasing burst pressure during our experiment. Although none of the LigaSure devices under test showed any loss of function, one Caiman instrument was damaged following the fourth sterilization procedure.

Our study has several limitations. The first one is due to the ex vivo approach. Although arteries were harvested directly from carcasses and kept cool and moist and all procedures took place within eight hours after slaughtering, effects of autolysis could not be ruled out. Additionally, postoperative complications, such as hemorrhage, could not be evaluated following this protocol. Another limitation is the number of devices tested. Four Caiman devices and 6 LigaSure devices performed 25 seals each, with half of the instruments undergoing a sterilization procedure after every fifth seal. For all seals performed with a MarSeal, only one instrument was used according to the manufacturer’s recommendation.

## Conclusion

During a limited number of cycles and sterilization procedures, seals created with the disposable vessel sealing device LigaSure achieved burst pressures similar to those produced with the reuseable MarSeal. One of the tested Caiman devices was damaged following sterilization. All seals produced during this study achieved supra-physiologic burst pressures. No seal failure was noted.

## Disclosure

Marseal and Caiman devices were funded by KLS Martin and Braun VetCare: KLS Martin Gmbh (DE): 1 x “marSeal 5plus” reuseable device, 10 x disposable blade carrier, Braun Vet Care (DE): 8 “Caiman 5” devices. This does not alter our adherence to PLOS ONE policies on sharing data and materials. The funders had no role in study design, data collection and analysis, decision to publish, or preparation of the manuscript.

## Supporting information

S1 Table Descriptive statistics and dependent variable: Burst pressure. Seal No. = Number of seal within one cycle, steril = Device underwent sterilization, Level = Powerlevel the device worked in, Type = Instrument type, Mean = Mean of messured values, Std. Deviation = Standard Deviation of messured values, N = Total number of seals.
(PDF)
