# Supplementary material for: Effect of multiple use and sterilization on sealing performance of bipolar vessel sealing devices
Source: PLoS One. 2019 Aug 20;14(8):e0221488. doi: 10.1371/journal.pone.0221488 (PMC6701801; doi:10.1371/journal.pone.0221488)
Supplement: S1 Table — Seal No. = Number of seal within one cycle, steril = Device underwent sterilization, Level = Powerlevel the device worked in, Type = Instrument type, Mean = Mean of messured values, Std. Deviation = Standard Deviation of messured values, N = Total number of seals. (PDF) [file pone.0221488.s001.pdf]

Seal No.	steril	Level	Type	Mean	Std. Deviation	N
1	No	2	MarSeal	537.00		1
		2	Caiman	715.00	257.39	2
		2	LigaSure	550.00		1
		2	Total	629.25	178.65	4
		3	MarSeal	1476.00		1
		3	LigaSure	1275.00	854.19	2
		3	Total	1342.00	615.05	3
		2+3	MarSeal	1006.50	663.97	2
		2+3	Caiman	715.00	257.39	2
		2+3	LigaSure	1033.33	734.86	3
		2+3	Total	934.71	534.91	7
	Yes	2	MarSeal	1897.00		1
		2	Caiman	877.00	66.47	2
		2	LigaSure	784.00		1
		2	Total	1108.75	528.72	4
		3	MarSeal	2215.00		1
		3	LigaSure	1234.50	617.30	2
		3	Total	1561.33	714.84	3
		2+3	MarSeal	2056.00	224.86	2
		2+3	Caiman	877.00	66.47	2
		2+3	LigaSure	1084.33	508.12	3
		2+3	Total	1302.71	607.15	7
	Yes+ No	2	MarSeal	1217.00	961.67	2
		2	Caiman	796.00	179.73	4
		2	LigaSure	667.00	165.46	2
		2	Total	869.00	446.29	8
		3	MarSeal	1845.50	522.55	2
		3	LigaSure	1254.75	608.92	4
		3	Total	1451.67	608.39	6
		2+3	MarSeal	1531.25	728.67	4
		2+3	Caiman	796.00	179.73	4
		2+3	LigaSure	1058.83	565.74	6
		2+3	Total	1118.71	582.37	14

Seal No.	steril	Level	Type	Mean	Std. Deviation	N
5	No	2	MarSeal	1909.00		1
		2	Caiman	696.50	316.08	2
		2	LigaSure	1336.00		1
		2	Total	1159.50	611.43	4
		3	MarSeal	1263.00		1
		3	LigaSure	1565.00	130.11	2
		3	Total	1464.33	197.14	3
		2+3	MarSeal	1586.00	456.79	2
		2+3	Caiman	696.50	316.08	2
		2+3	LigaSure	1488.67	161.07	3
		2+3	Total	1290.14	475.85	7
	Yes	2	MarSeal	1261.00		1
		2	Caiman	1028.00	192.33	2
		2	LigaSure	1386.00		1
		2	Total	1175.75	209.86	4
		3	MarSeal	2199.00		1
		3	LigaSure	1413.00	1139.86	2
		3	Total	1675.00	924.97	3
		2+3	MarSeal	1730.00	663.27	2
		2+3	Caiman	1028.00	192.33	2
		2+3	LigaSure	1404.00	806.15	3
		2+3	Total	1389.71	615.16	7
	Yes+ No	2	MarSeal	1585.00	458.21	2
		2	Caiman	862.25	286.82	4
		2	LigaSure	1361.00	35.36	2
		2	Total	1167.63	423.29	8
		3	MarSeal	1731.00	661.85	2
		3	LigaSure	1489.00	668.16	4
		3	Total	1569.67	609.17	6
		2+3	MarSeal	1658.00	472.34	4
		2+3	Caiman	862.25	286.82	4
		2+3	LigaSure	1446.33	522.00	6
		2+3	Total	1339.93	530.88	14

Seal No.	steril	Level	Type	Mean	Std. Deviation	N
6	No	2	MarSeal	918.00		1
		2	Caiman	621.00	26.87	2
		2	LigaSure	876.00		1
		2	Total	759.00	161.02	4
		3	MarSeal	1634.00		1
		3	LigaSure	847.50	61.52	2
		3	Total	1109.67	456.17	3
		2+3	MarSeal	1276.00	506.29	2
		2+3	Caiman	621.00	26.87	2
		2+3	LigaSure	857.00	46.51	3
		2+3	Total	909.29	342.72	7
	Yes	2	MarSeal	2381.00		1
		2	Caiman	821.50	234.05	2
		2	LigaSure	1550.00		1
		2	Total	1393.50	754.72	4
		3	MarSeal	948.00		1
		3	LigaSure	1218.00	429.92	2
		3	Total	1128.00	341.64	3
		2+3	MarSeal	1664.50	1013.28	2
		2+3	Caiman	821.50	234.05	2
		2+3	LigaSure	1382.67	359.39	3
		2+3	Total	1279.71	586.38	7
	Yes+ No	2	MarSeal	1649.50	1034.50	2
		2	Caiman	721.25	178.61	4
		2	LigaSure	1213.00	476.59	2
		2	Total	1076.25	608.48	8
		3	MarSeal	1291.00	485.08	2
		3	LigaSure	1032.75	329.59	4
		3	Total	1118.83	360.59	6
		2+3	MarSeal	1470.25	691.38	4
		2+3	Caiman	721.25	178.61	4
		2+3	LigaSure	1092.83	345.35	6
		2+3	Total	1094.50	499.85	14

Seal No.	steril	Level	Type	Mean	Std. Deviation	N
10	No	2	MarSeal	649.00		1
		2	Caiman	1191.50	549.42	2
		2	LigaSure	1557.00		1
		2	Total	1147.25	490.55	4
		3	MarSeal	1748.00		1
		3	LigaSure	1022.50	276.48	2
		3	Total	1264.33	462.25	3
		2+3	MarSeal	1198.50	777.11	2
		2+3	Caiman	1191.50	549.42	2
		2+3	LigaSure	1200.67	365.31	3
		2+3	Total	1197.43	442.11	7
	Yes	2	MarSeal	2384.00		1
		2	Caiman	658.50	301.94	2
		2	LigaSure	2781.00		1
		2	Total	1620.50	1136.04	4
		3	MarSeal	2015.00		1
		3	LigaSure	1401.50	207.18	2
		3	Total	1606.00	382.31	3
		2+3	MarSeal	2199.50	260.92	2
		2+3	Caiman	658.50	301.94	2
		2+3	LigaSure	1861.33	809.82	3
		2+3	Total	1614.29	833.26	7
	Yes+ No	2	MarSeal	1516.50	1226.83	2
		2	Caiman	925.00	475.09	4
		2	LigaSure	2169.00	865.50	2
		2	Total	1383.88	848.66	8
		3	MarSeal	1881.50	188.80	2
		3	LigaSure	1212.00	296.09	4
		3	Total	1435.17	423.39	6
		2+3	MarSeal	1699.00	746.99	4
		2+3	Caiman	925.00	475.09	4
		2+3	LigaSure	1531.00	668.32	6
		2+3	Total	1405.86	676.35	14

Seal No.	steril	Level	Type	Mean	Std. Deviation	N
11	No	2	MarSeal	502.00		1
		2	Caiman	814.50	135.06	2
		2	LigaSure	1515.00		1
		2	Total	911.50	435.50	4
		3	MarSeal	1800.00		1
		3	LigaSure	1930.00	190.92	2
		3	Total	1886.67	154.46	3
		2+3	MarSeal	1151.00	917.83	2
		2+3	Caiman	814.50	135.06	2
		2+3	LigaSure	1791.67	275.02	3
		2+3	Total	1329.43	611.95	7
	Yes	2	MarSeal	1735.00		1
		2	Caiman	651.00	65.05	2
		2	LigaSure	919.00		1
		2	Total	989.00	514.50	4
		3	MarSeal	1918.00		1
		3	LigaSure	1461.00	714.18	2
		3	Total	1613.33	569.77	3
		2+3	MarSeal	1826.50	129.40	2
		2+3	Caiman	651.00	65.05	2
		2+3	LigaSure	1280.33	594.09	3
		2+3	Total	1256.57	593.24	7
	Yes+ No	2	MarSeal	1118.50	871.86	2
		2	Caiman	732.25	128.07	4
		2	LigaSure	1217.00	421.44	2
		2	Total	950.25	443.22	8
		3	MarSeal	1859.00	83.44	2
		3	LigaSure	1695.50	505.46	4
		3	Total	1750.00	402.26	6
		2+3	MarSeal	1488.75	662.18	4
		2+3	Caiman	732.75	128.07	4
		2+3	LigaSure	1536.00	499.87	6
		2+3	Total	1293.00	580.26	14

Seal No.	steril	Level	Type	Mean	Std. Deviation	N
15	No	2	MarSeal	1302.00		1
		2	Caiman	838.00	90.51	2
		2	LigaSure	1796.00		1
		2	Total	1193.50	460.34	4
		3	MarSeal	1769.00		1
		3	LigaSure	1464.50	550.84	2
		3	Total	1566.00	427.34	3
		2+3	MarSeal	1535.50	330.22	2
		2+3	Caiman	838.00	90.51	2
		2+3	LigaSure	1575.00	433.98	3
		2+3	Total	1353.14	454.39	7
	Yes	2	MarSeal	15445.00		1
		2	Caiman	702.00	103.24	2
		2	LigaSure	2087.00		1
		2	Total	1259.00	682.77	4
		3	MarSeal	1849.00		1
		3	LigaSure	1731.00	434.16	2
		3	Total	1770.33	314.47	3
		2+3	MarSeal	1697.00	214.96	2
		2+3	Caiman	702.00	103.24	2
		2+3	LigaSure	1849.67	369.45	3
		2+3	Total	1478.14	583.74	7
	Yes+ No	2	MarSeal	1423.50	171.83	2
		2	Caiman	770.00	111.57	4
		2	LigaSure	1941.50	205.77	2
		2	Total	1226.25	540.22	8
		3	MarSeal	1809.00	56.57	2
		3	LigaSure	1597.75	433.18	4
		3	Total	1668.17	353.74	6
		2+3	MarSeal	1616.25	245.86	4
		2+3	Caiman	770.00	111.57	4
		2+3	LigaSure	1712.33	390.60	6
		2+3	Total	1415.64	506.73	14

Seal No.	steril	Level	Type	Mean	Std. Deviation	N
16	No	2	MarSeal	2028.00		1
		2	Caiman	713.50	205.77	2
		2	LigaSure	1245.00		1
		2	Total	1175.00	632.67	4
		3	MarSeal	2208.00		1
		3	LigaSure	1671.50	47.38	2
		3	Total	1850.33	311.56	3
		2+3	MarSeal	2118.00	127.28	2
		2+3	Caiman	713.50	205.77	2
		2+3	LigaSure	1529.33	248.51	3
		2+3	Total	1464,43	602.33	7
	Yes	2	MarSeal	1130.00		1
		2	Caiman	749.00	7.07	2
		2	LigaSure	1088.00		1
		2	Total	929.00	208.59	4
		3	MarSeal	1254.00		1
		3	LigaSure	1080.00	219.20	2
		3	Total	1138.00	184.71	3
		2+3	MarSeal	1192.00	87.68	2
		2+3	Caiman	749.00	7.07	2
		2+3	LigaSure	1082.67	115.07	3
		2+3	Total	1018.57	213.56	7
	Yes+ No	2	MarSeal	1579.00	634.98	2
		2	Caiman	731.25	120.62	4
		2	LigaSure	1166.50	111.02	2
		2	Total	1052.00	455.50	8
		3	MarSeal	1731.00	674.58	2
		3	LigaSure	1375.75	365.22	4
		3	Total	1494.17	452.44	6
		2+3	MarSeal	1655.00	542.02	4
		2+3	Caiman	731.25	120.62	4
		2+3	LigaSure	1306.00	306.88	6
		2+3	Total	1241.50	491.95	14

Seal No.	steril	Level	Type	Mean	Std. Deviation	N
20	No	2	MarSeal	724.00		1
		2	Caiman	1025.50	649.83	2
		2	LigaSure	1672.00		1
		2	Total	1111.75	548.15	4
		3	MarSeal	598.00		1
		3	LigaSure	1620.50	280.72	2
		3	Total	1279.67	622.82	3
		2+3	MarSeal	661.00	89.10	2
		2+3	Caiman	1025.50	649.83	2
		2+3	LigaSure	1637.67	200.72	3
		2+3	Total	1183.71	536.72	7
	Yes	2	MarSeal	1177.00		1
		2	Caiman	845.00	200.82	2
		2	LigaSure	2081.00		1
		2	Total	1237.00	595.43	4
		3	MarSeal	941.00		1
		3	LigaSure	1654.50	263.75	2
		3	Total	1416.67	452.19	3
		2+3	MarSeal	1059.00	166.86	2
		2+3	Caiman	845.00	200.82	2
		2+3	LigaSure	1796.67	308.90	3
		2+3	Total	1314.00	504.63	7
	Yes+ No	2	MarSeal	950.50	320.32	2
		2	Caiman	935.25	406.28	4
		2	LigaSure	1876.50	289.21	2
		2	Total	1174.38	534.04	8
		3	MarSeal	769.50	242.54	2
		3	LigaSure	1637.50	223.25	4
		3	Total	1348.17	492.53	6
		2+3	MarSeal	860.00	254.42	4
		2+3	Caiman	935.25	406.28	4
		2+3	LigaSure	1717.17	248.73	6
		2+3	Total	1248.86	504.81	14

Seal No.	steril	Level	Type	Mean	Std. Deviation	N
21	No	2	MarSeal	1492.00		1
		2	Caiman	697.00	224.86	2
		2	LigaSure	2771.00		1
		2	Total	1414.25	987.64	4
		3	MarSeal	1583.00		1
		3	LigaSure	1413.50	869.03	2
		3	Total	1470.00	622.24	3
		2+3	MarSeal	1537.50	64.35	2
		2+3	Caiman	697.00	224.86	2
		2+3	LigaSure	1866.00	995.93	3
		2+3	Total	1438.14	785.92	7
	Yes	2	MarSeal	2841.00		1
		2	Caiman	735.00		1
		2	LigaSure	1281.00		1
		2	Total	1619.00	1092.93	3
		3	MarSeal	1007.00		1
		3	LigaSure	1241.50	758.73	2
		3	Total	1163.33	553.32	3
		2+3	MarSeal	1924.00	1296.83	2
		2+3	Caiman	735.00		2
		2+3	LigaSure	1254.67	536.98	3
		2+3	Total	1391.17	813.97	6
	Yes+ No	2	MarSeal	2166.50	953.89	2
		2	Caiman	709.67	160.51	4
		2	LigaSure	2026.00	1053.59	2
		2	Total	1502.00	947.55	8
		3	MarSeal	1295.00	407.29	2
		3	LigaSure	1327.50	673.42	4
		3	Total	1316.67	552.77	6
		2+3	MarSeal	1730.75	782.16	4
		2+3	Caiman	709.67	160.51	4
		2+3	LigaSure	1560.33	790.07	6
		2+3	Total	1416.46	765.17	14

Seal No.	steril	Level	Type	Mean	Std. Deviation	N
5	No	2	MarSeal	1909.00		1
		2	Caiman	696.50		2
		2	LigaSure	1336.00		1
		2	Total	1159.50		4
		3	MarSeal	1263.00		1
		3	LigaSure	1565.00		2
		3	Total	1464.33		3
		2+3	MarSeal	1586.00		2
		2+3	Caiman	696.50		2
		2+3	LigaSure	1488.67		3
		2+3	Total	1290.14		7
	Yes	2	MarSeal	1261.00		1
		2	Caiman	1028.00		2
		2	LigaSure	1386.00		1
		2	Total	1175.75		4
		3	MarSeal	2199.00		1
		3	LigaSure	1413.00		2
		3	Total	1675.00		3
		2+3	MarSeal	1730.00		2
		2+3	Caiman	1028.00		2
		2+3	LigaSure	1404.00		3
		2+3	Total	1389.71		7
	Yes+ No	2	MarSeal	1585.00		2
		2	Caiman	862.25		4
		2	LigaSure	1361.00		2
		2	Total	1167.63		8
		3	MarSeal	1731.00		2
		3	LigaSure	1489.00		4
		3	Total	1569.67		6
		2+3	MarSeal	1658.00		4
		2+3	Caiman	862.25		4
		2+3	LigaSure	1446.33		6
		2+3	Total	1339.93		14

Seal No.	steril	Level	Type	Mean	Std. Deviation	N
25	No	2	MarSeal	1655.00		1
		2	Caiman	804.00	432.75	2
		2	LigaSure	829.00		1
		2	Total	1023.00	489.98	4
		3	MarSeal	944.00		1
		3	LigaSure	1217.50	416.49	2
		3	Total	1126.33	334.16	3
		2+3	MarSeal	1299.50	502.75	2
		2+3	Caiman	804.00	732.75	2
		2+3	LigaSure	1088.00	370.19	3
		2+3	Total	1067.29	400.39	7
	Yes	2	MarSeal	1692.00		1
		2	Caiman	737.00		1
		2	LigaSure	1775.00		1
		2	Total	1401.33	576.82	3
		3	MarSeal	1248.00		1
		3	LigaSure	986.00	552.96	2
		3	Total	1073.33	419.24	3
		2+3	MarSeal	1470.00	313.96	2
		2+3	Caiman	737.00		1
		2+3	LigaSure	1249.00	600.32	3
		2+3	Total	1237.33	485.46	6
	Yes+ No	2	MarSeal	1673.50	26.16	2
		2	Caiman	781.67	308.44	4
		2	LigaSure	1302.00	668.92	2
		2	Total	1185.14	521.39	7
		3	MarSeal	1096.00	214.96	2
		3	LigaSure	1101.75	421.43	4
		3	Total	1099.83	340.31	6
		2+3	MarSeal	1384.75	356.09	4
		2+3	Caiman	781.67	308.44	3
		2+3	LigaSure	1168.50	454.70	6
		2+3	Total	1145.77	431.44	13
